# A randomized, controlled study exploring factors associated with decision to undergo HIV screening

**DOI:** 10.1186/1471-2334-14-S2-P7

**Published:** 2014-05-23

**Authors:** Gerard O’Connor, Ailbhe Ni Flaitheartaigh, Aoife Lacey, Jane O’Halloran, Eamon Brazil, Yvette Calderon, Patrick WG Mallon

**Affiliations:** 1 HIV Molecular Research Group, School of Medicine and Medical Science, University College of Dublin, Dublin, Ireland

## Introduction

Little is known of the factors associated with a decision to test for HIV, particularly among varied gender and cultural groups. The Mater-Bronx Rapid HIV Testing (M-BRiHT) project explored if choice of pre-test counselor affected the decision to undergo HIV testing and hypothesized that offering expanded choice of counselor would lead to higher testing completion rates.

## Materials and methods

The M-BRiHT project is a single-site, prospective randomized study of adult Emergency Department attendees offered automated video-based pre-and post-test counseling combined with rapid HIV testing (buccal swab). Subjects were randomly assigned to receive identical, standardized video-based pre- and post-test counseling from a single pre-assigned counselor (Caucasian female) or to choose one of four counselors (male or female from Caucasian or African origin). Primary endpoint was the proportion of subjects completing HIV testing in each randomization group.

## Results

Of 6,000 subjects recruited from September 2012 to 2013, 2950 (49.1%) were randomized to choice of counselor. Mean (SD) age was 40.9 (16.4) yrs, 48.8% were female and 91.0%, 2.5% and 3.1% were of Caucasian, African or Asian ethnicity respectively.

4,919 (82.0%) completed HIV testing, with significantly higher completion rates in those randomized to choice of counselor (83.1 versus 80.9%, P<0.001). Other factors associated with higher HIV test completion rates included younger age (median 36 (SD 15.6) versus 46 (SD 18.6) yrs, P<0.001); and male gender (84.1 % v 79.8%, P< 0.001).

Fourteen subjects tested HIV positive (prevalence 2.8/1000), with 10 new HIV diagnoses and four re-confirmed HIV positive and re-linked to care. None had symptomatic HIV. Median (IQR) CD4+ T-cell count of new diagnoses was 515 (332, 595) cells/mm3 and all subjects were successfully linked to care.

## Conclusions

The M-BRiHT study demonstrates the feasibility of implementing large scale HIV screening within the Emergency Department and highlights the importance of offering choice of counselor when automated systems are employed to optimize HIV testing rates.

